# Future climate change and the distributional shift of the common vampire bat, *Desmodus rotundus*

**DOI:** 10.1038/s41598-025-87977-7

**Published:** 2025-02-18

**Authors:** Paige Van de Vuurst, Julia M. Gohlke, Luis E. Escobar

**Affiliations:** 1 Translational Biology, Medicine, and Health Program, Virginia Tech Graduate School, Blacksburg, VA USA; 2 Department of Fish and Wildlife Conservation, Virginia Tech, Blacksburg, VA 24061 USA; 3 Center for Emerging Zoonotic and Arthropod-borne Pathogens, Virginia Tech, Blacksburg, VA USA; 4 Department of Population Health Sciences, Virginia Tech, Blacksburg, VA USA; 5 Global Change Center, Virginia Tech, Blacksburg, VA USA

**Keywords:** *Desmodus rotundus*, Climate change, Ecological niche model, Rabies, Future climate models, Climate-change mitigation, Computational models, Infectious diseases

## Abstract

Interactions among humans, livestock, and wildlife within disturbed ecosystems, such as those impacted by climate change, can facilitate pathogen spillover transmission and increase disease emergence risks. The study of future climate change impacts on the distribution of free-ranging bats is therefore relevant for forecasting potential disease burden. This study used current and future climate data and historic occurrence locations of the vampire bat species *Desmodus rotundus*, a reservoir of the rabies virus to assess the potential impacts of climate change on disease reservoir distribution. Analyses included a comprehensive comparison of different climate change periods, carbon emission scenarios, and global circulation models (GCMs) on final model outputs. Models revealed that, although climatic scenarios and GCMs used have a significant influence on model outputs, there was a consistent signal of range expansion across the future climates analyzed. Areas suitable for *D. rotundus* range expansion include the southern United States and south-central portions of Argentina and Chile. Certain areas in the Amazon Rainforest, which currently rests at the geographic center of *D. rotundus*’ range, may become climatically unsuitable for this species within the context of niche conservatism. While the impacts of rabies virus transmitted by *D. rotundus* on livestock are well known, an expansion of *D. rotundus* into novel areas may impact new mammalian species and livestock with unexpected consequences. Some areas in the Americas may benefit from an assessment of their preparedness to deal with an expected *D. rotundus* range expansion.

**Supplementary Information:**

The online version contains supplementary material available at 10.1038/s41598-025-87977-7.

## Introduction

Climate change is a global driver of ecosystem degradation, which has been found to have chain and unexpected effects on ecosystem health^1–5^. Changes in global temperature, precipitation seasonality, and weather patterns have been well documented as a result of climate change, which can impact ecosystem stability and function^6–10^. The Intergovernmental Panel on Climate Change (IPCC) has predicted that climate change will amplify negative health impacts across the globe^1,6^. Ecosystem degradation linked to climate change includes distributional shifts for many wildlife species^11,12^. Models of species responses to climate change have predicted that various species will experience latitudinal and altitudinal shifts in their distributions^13–16^. It is anticipated that these distributional shifts may facilitate the creation of novel species assemblages^14,15,17^. Distributional shifts may also increase the transmission of pathogens to novel hosts^18^. Climate change can therefore act as a potential driver of disease emergence^18^. Pathogen spillover, or the transmission of a pathogen from one species to another, has been linked to some of the most lethal zoonotic diseases which impact humans^19^. Distributional shifts and poor wildlife health driven by climate change can increase risks of pathogen spillover from wildlife to humans via their disruption of multiple biological processes^20^. More specifically, spillover from wildlife to livestock has been shown to have negative impacts on animal and human health, and can cause considerable economic losses^19,21^.

Bats can tolerate infection by viruses that are highly pathogenic to livestock and humans^22,23^. Examples of high-impact spillover-derived zoonotic diseases which are transmitted by bats include Nipah virus encephalitis, severe acute respiratory syndrome (SARS), and rabies^24–26^. Currently, we lack a comprehensive understanding of how anthropogenic climate change may influence the ecology, behavior, or potential geographic distribution of bat disease reservoirs. Furthermore, climate change impacts on health are not evenly distributed geographically^27^, with some areas in the tropics being among the most impacted by emerging infectious diseases due to ecosystem degradation and climate change^10,27,28^. Low-income countries in the tropics are even more vulnerable to bat-borne emerging diseases due to compounding risk factors^29,30^. The study of future climate change impacts to the distribution of bats is, therefore, critically important for both human and animal health. Nevertheless, research in the tropics on this topic has been scarce to date^31^.

In Latin America, rabies virus (RABV) is a well-documented and impactful bat-borne pathogen^26,32^. Thousands of cattle are lost to bat-borne rabies in Latin America annually^33–35^, and billions of United States (US) dollars are forfeit annually for rabies prevention and control measures^36^. Rabies is almost 100% fatal for infected individuals, with dog rabies killing ~ 59,000 people a year, mainly in Africa and Asia^37,38^. Rabies has been reported to cause at least US$8.6 billion in economic losses in impacted areas due to loss of working hours of humans and death of their livestock^39^. The common vampire bat, *Desmodus rotundus*, is a common and widespread bat occurring across Latin America^40^. *Desmodus rotundus* feeds upon the blood of a variety of prey types including wildlife, livestock, pets, and humans^26^. During feeding, *D. rotundus* can transmit RABV to their prey^41^, thus acting as a wildlife reservoir and known host for the virus. Vampire bat-borne RABV (VB-RABV) outbreaks in humans and livestock regularly occur in tropical and subtropical regions^42^, where risks of human infection have been directly correlated with risks of outbreaks in livestock^34^.

Recent scientific literature has documented changes to *D. rotundus*’ distribution^43–45^. In the last 120 years, *D. rotundus* has expanded its range northward, towards the continental US due to climate change^44,46^. Furthermore, Benavides et al. (2016) documented an expansion of the geographic distribution of VB-RABV outbreaks in livestock in Peru, with a concurrent shift of *D. rotundus* populations into higher elevations. The expansion of *D. rotundus* and associated VB-RABV into southern portions of South America has also been linked to changes in the landscape and climate^43,47^. As such, there is sufficient evidence to suggest that future climate change may continue to shift the distribution of *D. rotundus* and potentially VB-RABV spillover. Ecological niche modeling can be used to assess the geographic distribution of a species fundamental or realized niche via computational assessment of species occurrence and environmental variables^48–50^. To assess the possible impacts of different climate change scenarios on the future distribution of *D. rotundus*, we evaluated multiple future climate change scenarios on the likely distribution of *D. rotundus’* fundamental niche^51^.

## Methods

We utilized ecological niche modeling-based methods to forecast the likely future distribution for *D. rotundus* across multiple future-climate scenarios in the Americas. We used MaxEnt^52^, which functions by comparing the environmental conditions of occurrence locations with those of selected background locations from the available environmental space where the species could potentially occur (i.e., environmental “similarity” between field observations and study area)^52–54^. MaxEnt is a frequently utilized presence-background modeling platform successfully employed in previous assessments of *D. rotundus*’ distribution^44,46,55^. MaxEnt does not require true absence data for its calibration, and therefore more logically abides by the available occurrence locations data for *D. rotundus*^56^. We chose to make a correlative assessment of *D. rotundus*’ fundamental niche using only abiotic climate data as background environmental variables, rather than incorporating biotic or movement variables to assess the realized niche^50,51,57^. While biotic variables have been shown to benefit ecological niche models at finer spatial scales^58^, our assessment was conducted across a larger spatial scale and was focused on projections of future climate. We chose to mitigate the potential impacts of uncertainty from non-climate variables such as future land-use projections^59^ or future cattle densities to glean a more comprehensive understanding of future climate variability alone.

### Occurrence location filtering

**Fig. 1:**
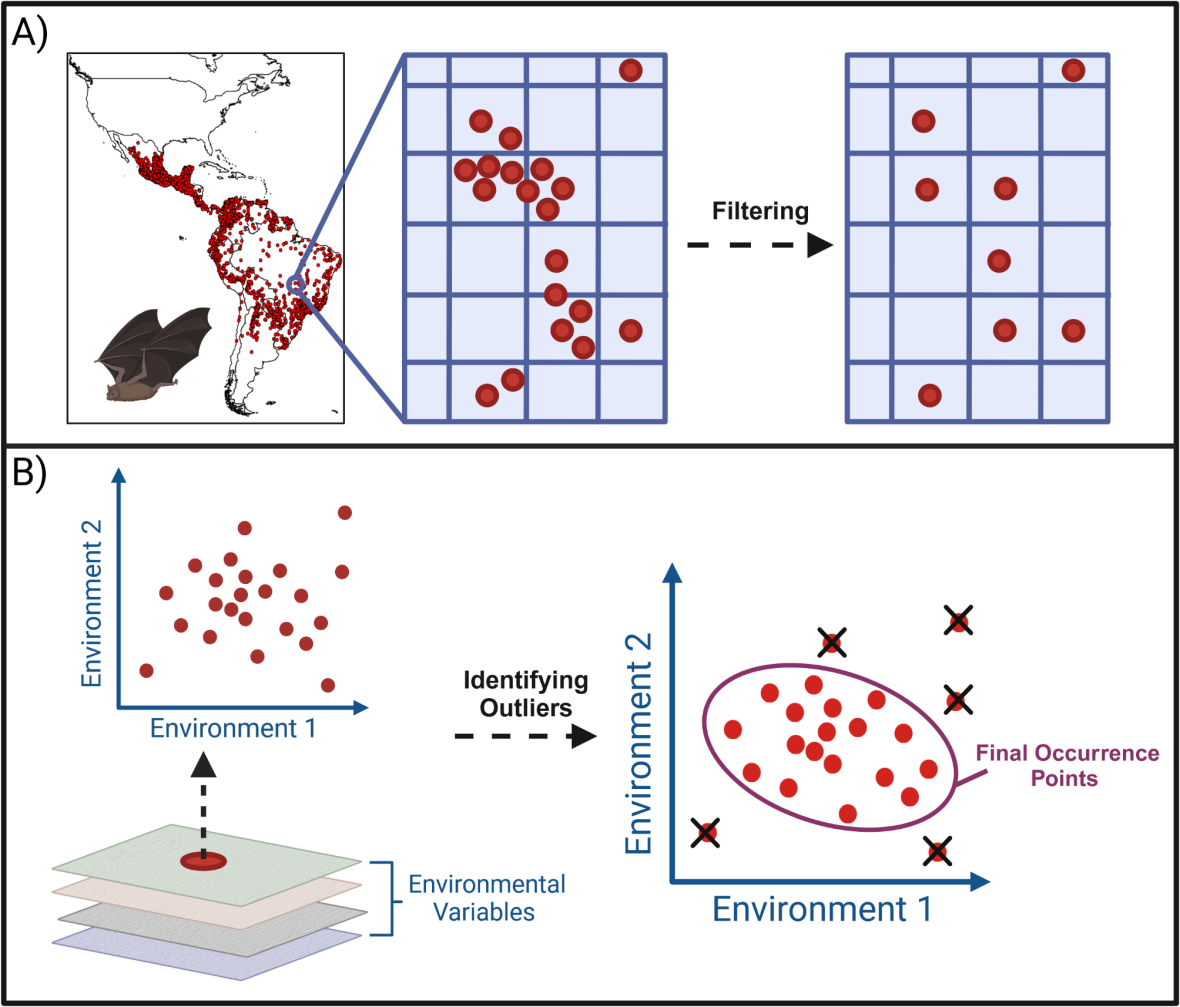
Occurrence Location Filtering. **A** Geographic Space: Filtering of *Desmodus rotundus* occurrence locations from 1900-2023^60^ in geographic space. Occurrence locations were resampled to one per pixel (2.5 arc-minute or ~ 5 km resolution) of the study area to reduce the overrepresentation of certain environmental conditions. **B** Environmental Space: Values of background environmental variables averaged from years 1970–2000 were extracted from the location of geographically filtered occurrence locations. These data were then used to create an environmental space^64^ where we calculated the distance between occurrence locations using Mahalanobis distance. Using a chi-squared test, we identified environmental outliers via drawing an ellipsoid, where occurrence location which fell outside the ellipsoid (*n* = 269 or 4.6% of the occurrence locations) were excluded from final occurrence locations used for our modeling effort. Created in BioRender. Van de Vuurst, P. (2025) https://BioRender.com/z34g286.

We collected occurrence locations of *D. rotundus* from an extensive database specific to this species from across Latin America from 1901 to 2023^60^. We isolated all occurrence locations from this database with associated location data (i.e., geographic coordinates or locality descriptions) using the *dplyr* package in R statistical software version 4.1.0 resulting in 74.6% geolocated records (*n* = 29,174 of 39,118 available records)^61^. To address possible sample selection bias and spatial autocorrelation within the occurrence locations data, we filtered the *D. rotundus* occurrence locations to one per pixel (Fig. 1). Pixels were delineated based on the background environmental variables used for model calibration at 2.5-arc-minute (~ 5 km) resolution. To resample the occurrence locations to one per pixel, we created a blank raster mask of the same spatial scale and resolution as the background environmental variables for which each pixel was assigned a pixel index value for its corresponding location within the study area (i.e., Latin America). We then removed all but one occurrence location for each pixel index value within the study area (*n* = 5788). As the current estimates of *D. rotundus* climatic suitability were to be calibrated using current climate estimates averaged across 30 years (1970–2000), occurrence locations that were redundant by location (i.e., duplicate coordinates) were not retained, even if they were collected in different years. This allowed us to mitigate any overrepresentation of certain oversampled climatic conditions.

The remaining occurrence locations were then matched with the corresponding values of the background environmental variables (i.e., climate) from the WorldClim database^62^ at 2.5-arc-minute resolution (~ 5 km). WorldClim provides 19 different global metrics of climate, both for current climates and for a variety of different future periods and emissions scenarios^62^. Current climate data from WorldClim are averaged from the years 1970-2000^62^, which were used as the closest approximation of the available historical occurrence location data. We used the *raster* package^63^ in R to extract the value of each WorldClim variable for current climates at the location of each occurrence location. We used these values to create a cloud of data points representing the species historic distribution in environmental space (E-space)^64^. We then developed a principal component analysis of the background environmental variables (i.e., current climate) extracted at each occurrence location to obtain principal component axes summarizing variance of the climatic data at location of known occurrence. Principal components one, two, and three (summarizing 80.4% of the background environmental variable data variance) were then used as axes to plot the occurrence locations in environmental space. We then calculated the Mahalanobis distances between each occurrence location in E-space. These distances were used to fit a minimum-volume ellipsoids to all occurrence locations in E-space with permutations taking one point out in each iteration. A Chi-squared test was used to identify which occurrence locations fell outside of the ellipsoid. Occurrence locations significantly outside the bulk of ellipsoids (*n* = 269 or 4.6% of the occurrence locations) were identified as environmental outliers (i.e., occurrence locations which do not represent the “typical” climate tolerances of *D. rotundus*) and were removed (following methods of Qiao et al. 2024). After filtering in geographic and environmental space, the remaining 5519 occurrence locations were randomly split into 50% training 50% testing subsets for model calibration and evaluation.

### Ecological niche modeling

Ecological niche model calibration, evaluation, and projection was completed in R using the *kuenm* package^65^. While 19 different metrics of climate are available within the WorldClim database, some of these variables are correlated. To reduce background environmental variable redundancy, we used a Pearson Correlation coefficient analysis^64^ of all 19 variables to eliminate highly correlated variables (i.e., *ρ* ≥ 0.5, *n* = 12). The remaining seven climate variables were evaluated based on their known importance to the ecology of *D. rotundus*. As such, three climatic variables (mean diurnal temperature range, maximum temperature of the warmest month, and precipitation of the driest quarter) were eliminated from consideration. Four final climatic variables were used as background environmental variables, including precipitation seasonality (standard deviation of monthly precipitation estimation as a percentage of annual mean precipitation) (kg m^− 2^), annual precipitation (kg m^− 2^), minimum temperature of the coldest month (°C), and isothermality (mean diurnal temperature range divided by annual temperature range) (°C). We then restricted the background environmental variables to a specific calibration area^64^. To create this calibration area we used ArcGIS Pro version 2.5 software^66^ to create a 200 km buffer around each occurrence location, which is ten times the species’ home range^67,68^ and encompasses any amount of long range dispersal that the species may have experienced in current climates^43,69^. This buffer was exported as a shape file and used to crop the background environmental variables to the calibration area. By limiting the calibration area in this way, areas that the species may not have access to due to dispersal limitations are reduced from consideration during the calibration process, thus making the comparison between presence and background more accurate^64^.

The *kuenm* package allowed us to create candidate models with multiple parameterizations and feature class assumptions in MaxEnt^70^. MaxEnt feature classes are functions derived from background environmental variables to minimize model overfitting, and regularization multipliers are parameters which impose penalties on models for over-complexity^71,72^. We tested a suite of regularization parameters that modulate model fit to the data (i.e., 0.1, 0.2,0.5,1,2, and 5) and all possible combinations of five feature classes that dictate the assumed species responses to the environmental variables (linear, quadratic, product, threshold, and hinge). After redundant model combinations were removed, the remaining 434 candidate models were evaluated based on omission rates (*E* = 0.05)^73^ and model complexity (*AICc*)^65^. Evaluation was conducted using the kuenm_ceval function in *kuenm* package^65^.

The final ecological niche model selected from the evaluation process was then projected to the geographic extent of the study area (the Americas) and to multiple future climate change scenarios across six Global Circulation Models (GCMs). These GCMs included the Australian Community Climate and Earth System Simulator (ACCESS-CM2), the Beijing Climate Center Climate System Model (BCC-CSM2-HR), the UK Earth System Model (UKESM1-0-LL), the Europe wide consortium climate model (EC-Earth3-Veg-LR), the National Institute for Environmental Studies of the University of Tokyo model (MIROC6), and the Max Planck Institute for Meteorology Model (MPI-ESM1-2-LR) (Table 1). These six models were chosen to capture GCM-projected climate variability as available in the most recent iteration of NEX-GDDP CMIP6 GCMs^74–76^. These six models also capture equilibrium climate sensitivity (ECS) and transient climate response (TCR) variance from high to low^75^ (Table 1). Both ECS and TCR are standard metrics of climate model sensitivity related to increased CO_2_ concentrations^75^.

**Table 1:** Global circulation model sensitivity.

Global Circulation Model Name	Abbreviation	ECS	TCR
Australian Community Climate and Earth System Simulator	ACC	4.7	2.1
Beijing Climate Center Climate System Model	BCC	3	1.7
Europe Wide Consortium Climate Model	EVeg	4.3	2.6
National Institute for Environmental Studies, University of Tokyo Model	MIROC	2.6	1.6
Max Planck Institute for Meteorology, Germany Model	MPI	3	1.8
United Kingdom Earth System Model	UK	5.3	2.8

Equilibrium climate sensitivity (ECS) and transient climate response (TCR) metrics from each associated Earth system global circulation model (GCM) as reported in Meehl et al. (2020)^75^. Both ECS and TCR are standard metrics of climate model sensitivity related to increased CO_2_ concentrations. These six models were chosen to capture GCM-projected climate variability as available in the most recent iteration of NEX-GDDP CMIP6 GCMs^74–76^. These six models also capture ECS and TCR variance from high to low^75^.

**Fig. 2:**
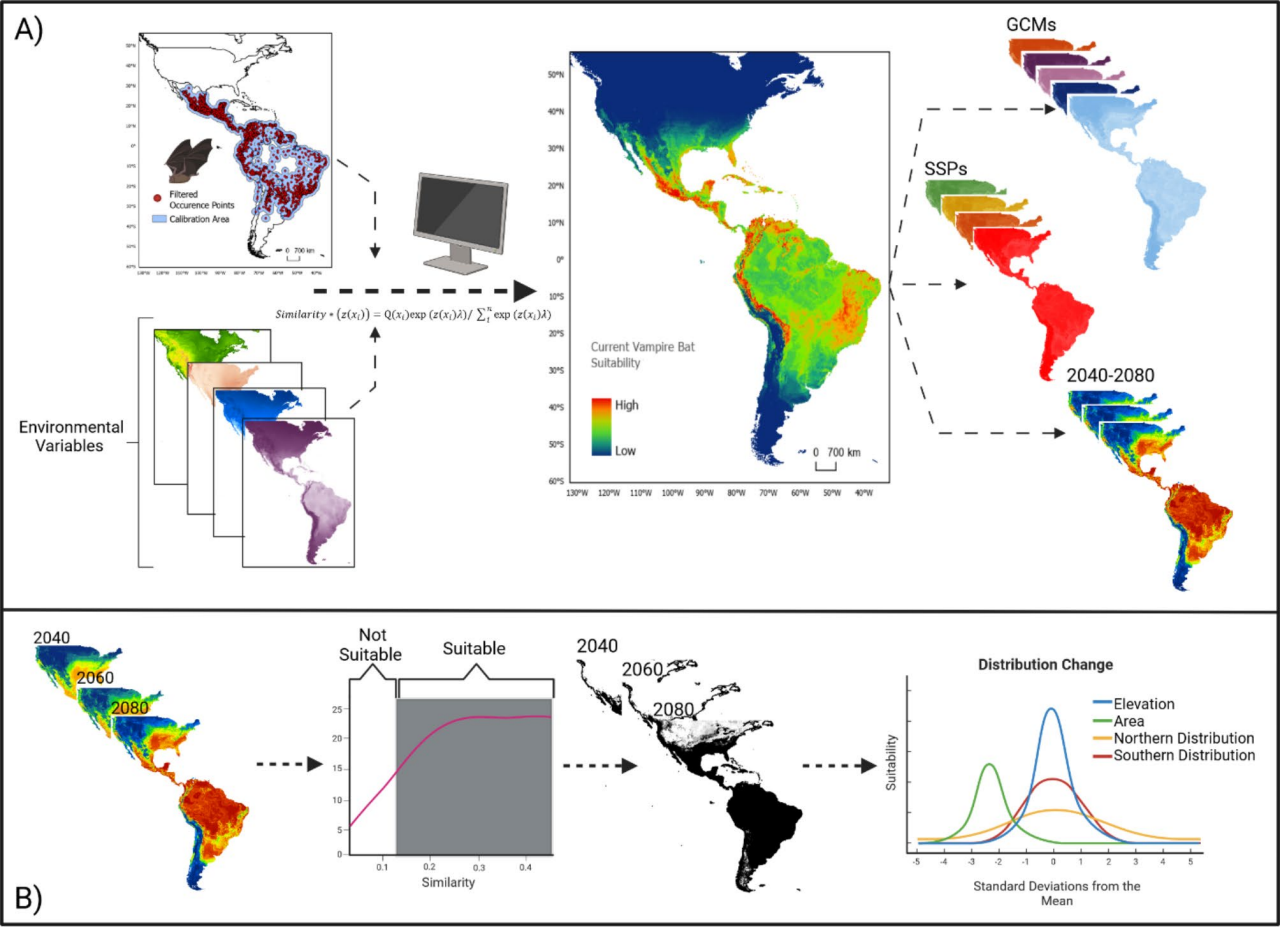
Model calibration and pattern analysis. **A** Model calibration: We used filtered occurrence locations and environmental variables from the WorldClim database^62^, with a calibration area of 200 km^2^ (ten times species home range)^67,68^ around the occurrence locations to calibrate MaxEnt models. The best model was used to estimate current suitability for *Desmodus rotundus*. We then projected suitability to future climate change projections across six global circulation models (GCMs), four possible future shared socio-economic pathways (SSPs one, two, three, and five), and three time periods (2040, 2060, and 2080). **B** Potential future distributions: We used a minimum training presences threshold to isolate areas with suitable climates for *D. rotundus* based on our model’s projections. We used this threshold to create binary rasters of future scenarios to explore possible changes in maximum and minimum latitude of projected occurrence (i.e., range shift north or south), total area (km^2^) predicted as suitable for occurrence, and maximum projected elevation (i.e., range shift up mountain ranges into previously temperate areas). Created in BioRender. Van de Vuurst, P. (2025) https://BioRender.com/z34g286.

We also projected the final ecological niche model across four possible future shared socio-economic pathways (SSPs one, two, three, and five), which capture variation in the magnitude or severity of climate change based on possible future global greenhouse gas emissions and future population growth and development trajectories^77,78^. Each GCM and SSP was used as a reference climate to project the final ecological niche model across three different time periods, including 2020–2040, 2041–2060, and 2061–2080. This combination of GCMs, SSPs, and time periods allowed us to develop a comprehensive assessment of likely *D. rotundus* distributions across a variety of possible future climates, and captures variation as a proxy of uncertainty in the species’ future distribution (Fig. 2).

The continuous log MaxEnt model outputs (i.e., clog outputs) were interpreted as a numeric representation of the similarity of the projected location to the bulk of locations with known occurrences of *D. rotundus* (i.e., the training data) from the calibration area^54,79^. These values can also be theoretically interpreted as a metric of how “suitable” each location is for *D. rotundus* occurrence relative to the background environmental variables (i.e., in this case, climate in the Americas).

MaxEnt models can be projected using free extrapolation, limited extrapolation, or no extrapolation to predict suitability in environments outside values from the occurrence locations^65,80^. Extrapolation would allow features to extend beyond the ranges of the training data, predicting into novel climatic conditions not currently available in the model-calibration area^79^. In other words, projecting MaxEnt models with free or limited extrapolation would assume that the species has physiological tolerances beyond the tolerances observed under current climates. Alternatively, model projection using extrapolation assumes that the species would evolve fast to adapt to novel climates^81^. Thus, model extrapolation violates ecological niche conservatism, which postulates that species are expected to retain climate tolerances under rapid climate change^81^. Within the *kuenm* package we therefore chose to project our model to future climates without extrapolation, limiting the uncertainty of our projection into future time periods^65^. The strict model transferences without extrapolation allowed us to make inferences and conclusions from a comparatively conservative representation of projected future suitability for *D. rotundus*.

### Post projection analysis

After model projection, we utilized *kuenm* post-projection processing functions to identify areas of strict extrapolation risk via a mobility-oriented parity (MOP) analysis for all four SSPs for the 2080 time period. A MOP analysis identifies areas with the most dissimilar climate conditions from the background environmental variables in the calibration data (i.e., where one or more covariate variables are outside ranges present in the training data). This analysis allowed us to identify areas where extrapolation could impact the certainty of future suitability projections. We summarized these results by summing all four MOP rasters into one map across all SSPs to visualize geographic areas with strict extrapolation risk, regardless of projected climate change severity.

The projected continuous suitability maps created during model projections were ensembled for each time period and SSP by adding them using the *raster* package in R version 4.1.0^56^. The model ensemble allowed us to identify how the magnitude of climate change may impact the distribution of *D. rotundus* across time, regardless of GCM. We also used these GCM ensembles to assess changes in suitability for *D. rotundus* across time periods (Fig. 2).

Additionally, we quantified the directions and magnitudes of any *D. rotundus* distributional changes projected by using a minimum training presences threshold to isolate areas suitable for *D. rotundus* based on the final ecological niche model. This minimum training presence value was used as a threshold to reclassify the continuous projected maps into binary maps. Model binarization was based on the assumption that the least suitable location at which the species is known to have occurred is the minimum suitability value for the species^82^. The resulting binary maps were used to assess how the distribution of projected suitability for *D. rotundus* may change across time (Fig. 2).

We used the *raster* package^63^ to quantify the total suitable area in km^2^ from each binary map by SSP. We then isolated the 100 highest (most northern) and lowest (most southern) latitudes predicted by the projected range models by time period, which allowed us to assess whether or not the projected suitable range for *D. rotundus* may vary latitudinally based on time period or SSP. We also identified the 100 highest projected suitable elevations from each binary map by GCM and averaged across SSPs, to determine the extent to which *D. rotundus*’ potential range varied in elevation. Elevation data were collected from the WorldClim database at ~ 5 km spatial resolution in agreement with the climate data^62^. We used multiple regressions and two-way analysis of variance to assess trends in distributional ranges across the periods.

## Results

**Fig. 3:**
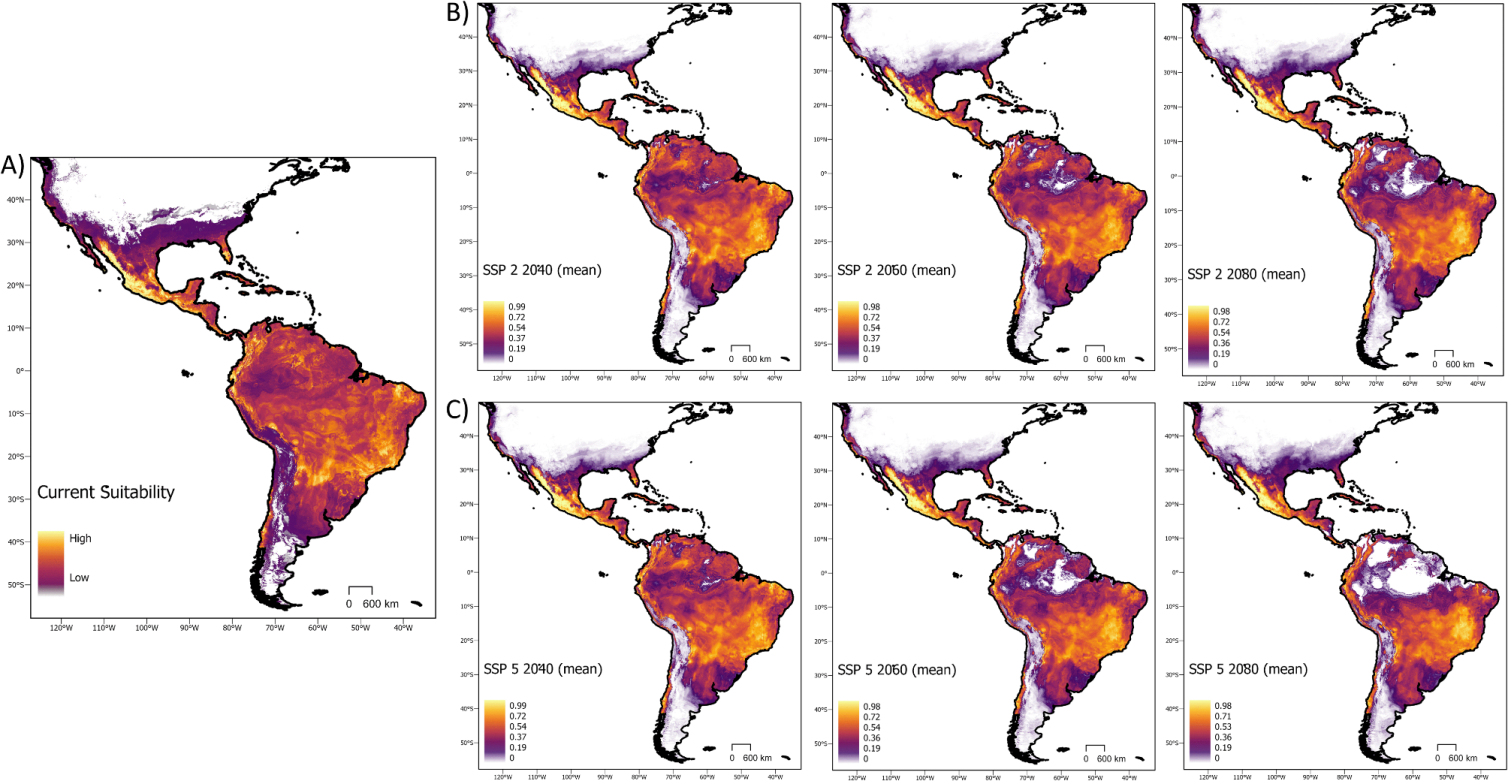
Suitability projections. Continuous log output of projected suitability from best MaxEnt model for current conditions and averaged across all six GCMs for future scenarios. SSP two (upper panel), and SSP five (lower panel) are shown as the most likely best case (SSP 2) and worst case (SSP 5) scenarios. Current suitability is shown for no suitability (below minimum training presence) (white), low suitability (purple), and high suitability (yellow). Suitability is show from low (purple) to high (yellow) for future scenarios in panels at right. Note the loss of suitable area in central portions of the Amazon rainforest in both 2060 and 2080 projections. SSP five, which anticipates greater changes to precipitation seasonality, had much more extreme changes to *D. rotundus* potential future distribution when compared to current estimates. Suitability for the species extended both northward and southward of current estimates. Figure created using ArcGIS Pro version 2.5 software^66^.

**Fig. 4:**
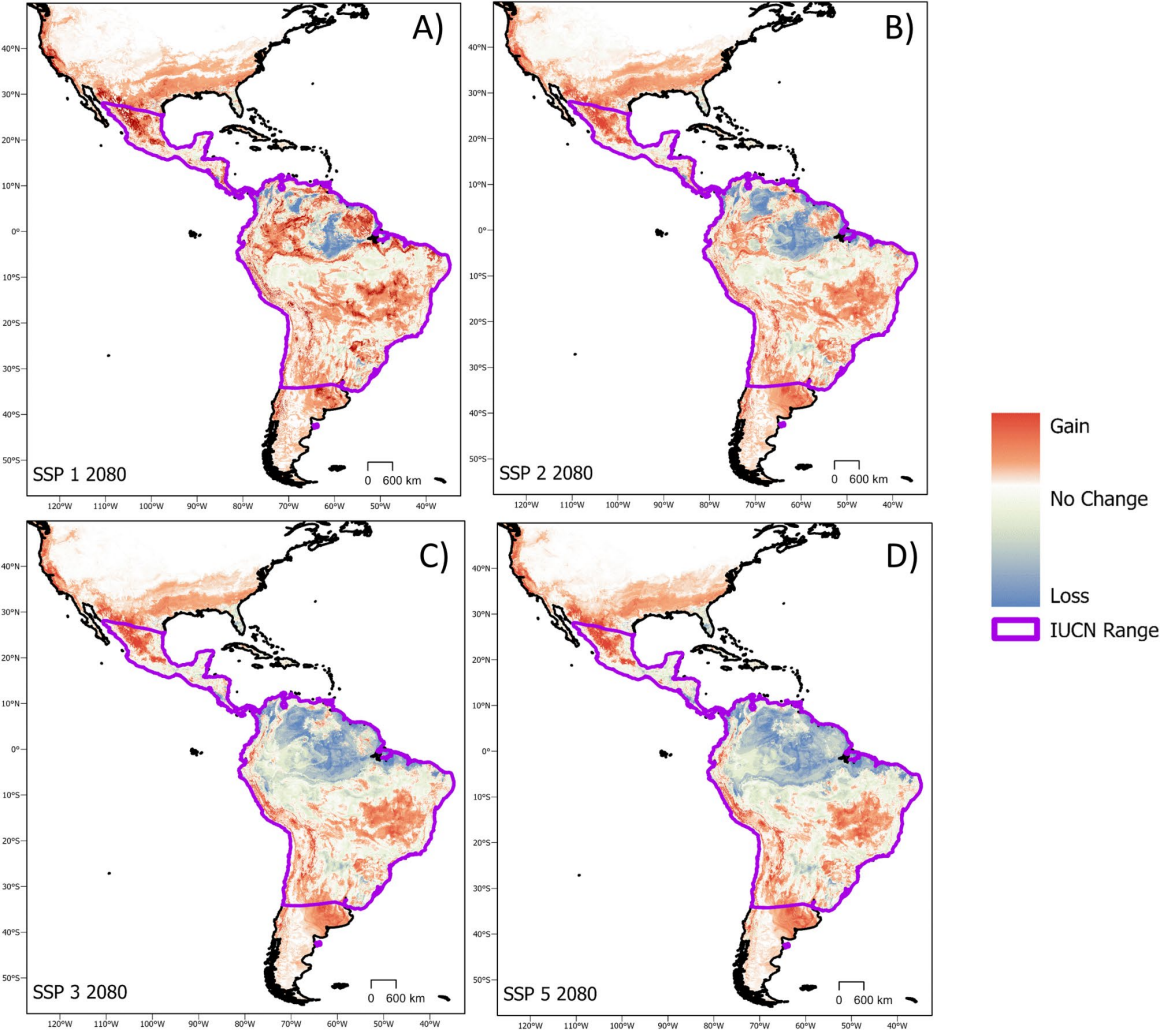
Change in future suitability. Continuous comparison of projected future suitability from the most distant future (2080) averaged across all GCMs to current estimates. Different shared socioeconomic pathways (climate change scenarios) are shown in panels **A** SSP1, **B** SSP2, **C** SSP3, and **D** SSP4. Areas with increase suitability are shown in red, areas with a loss of suitability are shown in blue. The current International Union for Conservation of Nature (IUCN) range for *D. rotundus* is shown in purple^40^. As future scenarios increase in their climate change impact, the loss of suitable area for *D. rotundus* in the central Amazon becomes more widespread. Increases in suitability under all SSPs are shown in western portions of Mexico, southern portions of the United States, and central portions of Argentina. Gains in suitability extended both northward and southward of current estimates. Figure created using ArcGIS Pro version 2.5 software^66^.

Of all 434 candidate ecological niche models, 304 performed better than random in terms of prediction of independent, testing data, and had omission rates lower than our exclusion threshold (*E* = 0.05). Final ecological niche model parameterizations and performance metrics were obtained from the model evaluation process. The most well-preforming model selected during the evaluation process had a regularization multiplier of 0.1 and used the linear, product, and threshold feature classes. The final ecological niche model omission rate was 0.048 and the overall AICc was 153,639.9 (Delta AICc = 0, Weighted AICc = 0.5). Minimum temperature of the coldest month had the highest variable contribution (32.7%), followed by precipitation seasonality (32.6%), annual precipitation (18.5%), and isothermality (16.1%). Based on the projection of this model, we found that *D. rotundus* is likely to extend its range both northward and southward of current range estimates in the next 20–80 years (Figs. 3 and 4). GCMs with lower sensitivity values of ECS had higher rates of variance in our distributional projections, specifically for our northern latitudinal analysis (Fig. 6). The Max Planck Institute model (MPI) and the Europe wide consortium model (EVeg) in particular had wider variance in the most northern projected suitable latitudes for all SSPs (Fig. 6).

**Fig. 5:**
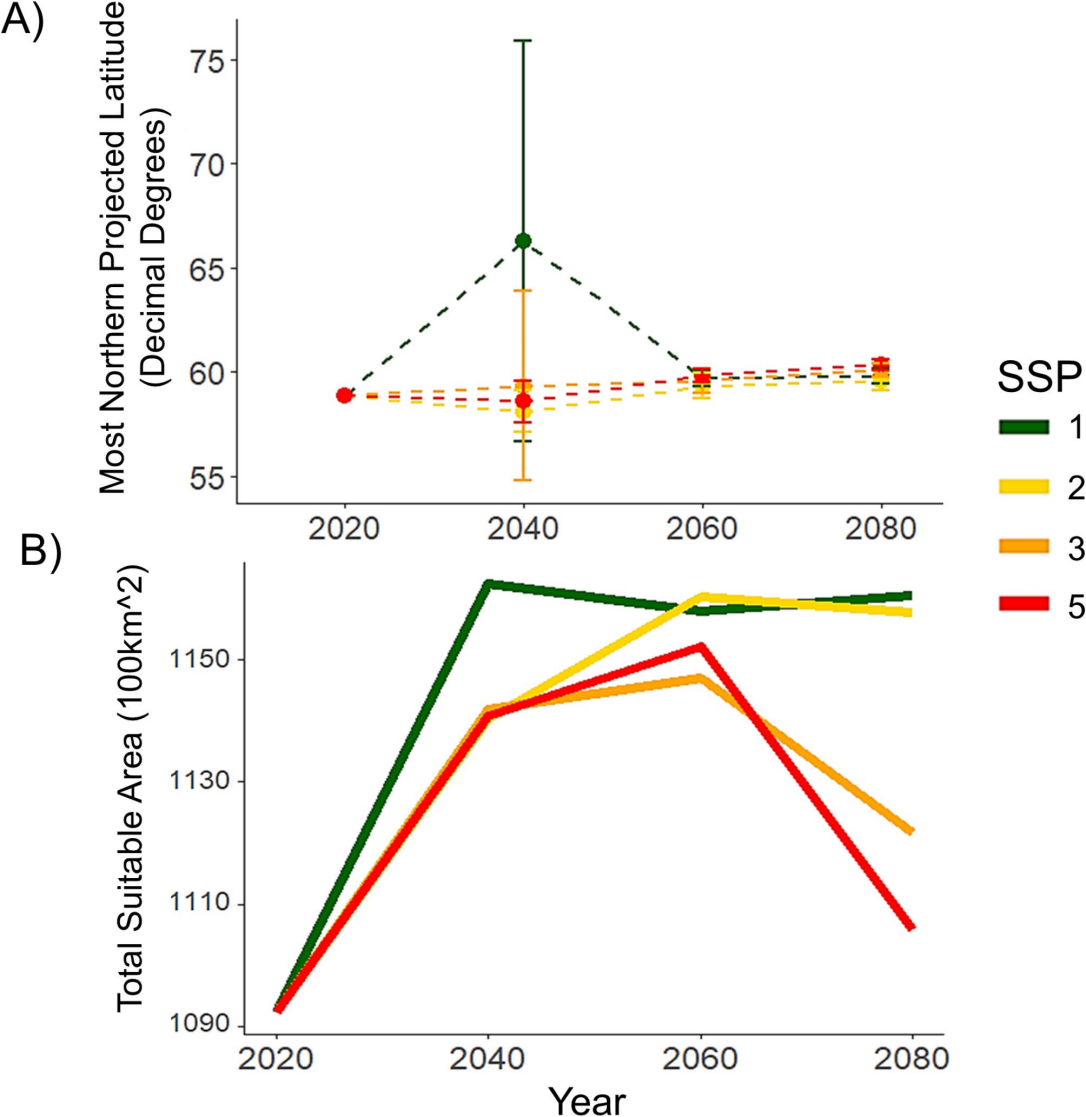
Projected suitability changes for*Desmodus rotundus*. **A** Averaged most northern predicted range (i.e., maximum latitudes predicted) and from binarized outputs of suitability for *D. rotundus* across all six GCMs. Error bars represent standard error. Each individual SSP showed increases in most northern suitable latitude. There was a significant increase in most northern projected suitable latitude for *D. rotundus* across all SSPs (*p* = 0.001, standard error = 3.48, R^2^ = 0.1). **B** Total suitable area identified from binarized outputs of suitable area for *D. rotundus* across all six GCMs. After including SSP as a random effect, there was a significant increase in total suitable area (*p* = 0.028, Standard Error = 276.9, R^2^ = 0.43) for *D. rotundus’* range across all projections. There were notable bell curve patterns in total suitable area for SSPs 3 and 5 (i.e., worst case scenarios).

Based on the average ensemble of all GCMs, a multiple linear regression model (Maximum Latitude ~ Period + SSP) showed that northern expansion was significant but modest across all SSP scenarios (*p* < 0.01, Adjusted R^2^ = 0.1, Standard Error = 3.48) (Fig. 5). Northern suitability peaked during the 2020–2040 period under the best-case scenario for sustainable development (SSP1). By 2080, northern expansion of suitability for *D. rotundus* was approximately two degrees of latitude (over 200 km) higher in the worst-case scenario of future climate (SSP 5). Southern expansion beyond current estimates of suitable range for *D. rotundus* existed across all SSPs, but was not significant per a multiple linear regression analysis (Minimum Latitude ~ Period + SSP) (*p* = 0.98, Adjusted R^2^ = 0.04, Standard Error = 0.13).

In terms of total suitable area, a linear model of all SSP across all GCMs (i.e., Total Suitable Area ~ period) denoted a significant increase in total suitable area (*p* = 0.028, Standard Error = 276.9, R^2^ = 0.43) from current projections to the 2080 time period. Nevertheless, there was no significant change in total suitable area for *D. rotundus* across the projected time periods when SSP was included as a predictor (Total Suitable Area ~ Period + SSP) (*p* = 0.15, Adjusted R^2^ = 0.22, Standard Error = 24770) (Fig. 5). Increases in total suitable area were maintained in later periods (2060 and 2080) of best-case scenarios (SSP 1 and 2). In later periods of future climate there was a decrease in total suitable area in the worst-case scenarios (SSPs 3 and 5) (Fig. 5). The distribution of suitable area for each scenario and time period was not consistent across the Americas. We found that certain areas in the Amazon, which currently rests at the geographic center of *D. rotundus*’ range^40^, may become unsuitable under more extreme climate change scenarios such as SSP 3 and 5 (Fig. 5). In these scenarios, the Amazon is expected to become drier and precipitation seasonality is expected to become more unstable^83,84^. In contrast, some areas that are currently unsuitable for *D. rotundus* occurrence are projected to gain climate suitability, including the southern US, western Mexico, and central Argentina (Fig. 5). Per the MOP analysis, areas with strict extrapolation risk in the GCMs corresponded with many of the areas projected to lose suitability (Fig. 4). The MOP strict extrapolation was located in the Amazon rainforest and in some areas of Central America (Fig. S1). As such, projections of future suitability for *D. rotundus* in these geographic regions may be more uncertain. We found no identifiable relationship between average suitable elevations for *D. rotundus* and time period or SSP (two-way ANOVA, *p* = 0.27). There was an increase of 30 m of average maximum elevation of suitability from current projections in the SSP 5 climate.

## Discussion

This study aimed to assess the impacts of future climate on estimates of the fundamental niche and potential distribution of a bat that serves as a disease reservoir. We used current and future climate data and historic occurrence locations of the rabies reservoir *D. rotundus* to reconstruct the distribution of this species under different climate change scenarios. We conducted a comprehensive comparison of the methodological implications of different climate change projections, including differing carbon emissions scenarios, time periods, and GCMs. We identified the potential for *D. rotundus* to expand its distribution into novel areas both north and south of current estimates. Models revealed areas that may lose or gain climate suitability for *D. rotundus* under different socioeconomic pathways and emissions scenarios (i.e., SSP). This study provides evidence of a potential range shift for *D. rotundus* due to future climate change.

Of the climate variables used as background environmental variables, minimum temperature of the coldest month, and precipitation seasonality had the highest contribution in the final model. These results echo previous modeling efforts^85,86^, which found minimum temperatures and seasonality to be major drivers of *D. rotundus* distribution. Previous research has suggested that *D. rotundus* may be poorly adapted to survival at lower temperatures^87^, as it is assumed that the species cannot effectively thermoregulate in colder climates due to its diet of only blood^88,89^. Nevertheless, a substantial degree of variation in the individual thermal tolerances has been observed among *D. rotundus* individuals^90^. The importance of precipitation seasonality in the ecological niche model could derive from the high annual precipitation across tropical and sub-tropical belts of the Americas^40,91^. For instance, some of the highest precipitation seasonality indices can be found in the Amazon rainforest^83,91^, which currently rests at the geographic center of *D. rotundus*’ range. Dry or desert areas have also been identified as barriers to dispersal for *D. rotundus*, especially at the Northern extent of its range in Mexico^46^. These currently-unsuitable areas could become suitable for *D. rotundus* occurrence, and thus no longer act as barriers to the species’ dispersal owing to variations in rain patterns due to climate change.

Climate suitability for *D. rotundus* was predicted to be lost in central portions of the Amazon rainforest. The Amazon is projected to experience compounding climate stressors and decreases in climate stability under future climate change scenarios^83,84^. The final ecological niche model predicted *D. rotundus* range expansion into novel areas such as western Mexico, southern US, and central Argentina. These regions of expansion may benefit from an assessment of their current preparedness to deal with a natural invasion of *D. rotundus* and the associated VB-RABV they may carry. Nevertheless, a potential invasion of *D. rotundus* does not completely necessitate an increase of pathogen spillover, as the presence of the reservoir does not wholly preclude the expansion of VB-RABV^47,92,93^. For example, in some disease systems there is evidence to suggest that the thermal optimum for hosts is different from that of their associated pathogens^94,95^. More research on the impacts of biotic or movement-based factors would need to be conducted at finer scales in areas predicted to gain suitability for *D. rotundus* to fully ascertain the risks of VB-RABV expansion^48,51,82^. Preparedness against VB-RABV prevention is especially important for areas predicted here to be suitable for *D. rotundus* with high livestock densities.

Areas of projected suitability gain in central Argentina and western Mexico have higher livestock densities (> 100 head of livestock per square kilometer) than areas of projected loss such as the Amazon (< 10 head of livestock per square kilometer)^96^. Loss of suitable area in our projections also corresponded to areas of known low human population density in countries such as Brazil, where densities are as low as < 1 person per square kilometer^97^. Areas of gain in suitability such as western Mexico and the southern US, in contrast, have population densities > 25 people per square kilometer, even in rural areas^97^. As such, large areas predicted to become climatically suitable for *D. rotundus* may host higher vulnerability to VB-RABV than current hotspots of *D. rotundus* suitability (Figs. 3 and 4)^96–98^. Greater conflict between *D. rotundus* and humans in these regions may drive greater persecution for this and other bat species via culling, which has not been proven to decrease spillover rates for RABV^47,99^. A climate change-driven range shift for *D. rotundus* may therefore exacerbate human-wildlife conflicts involving bats.

**Fig. 6:**
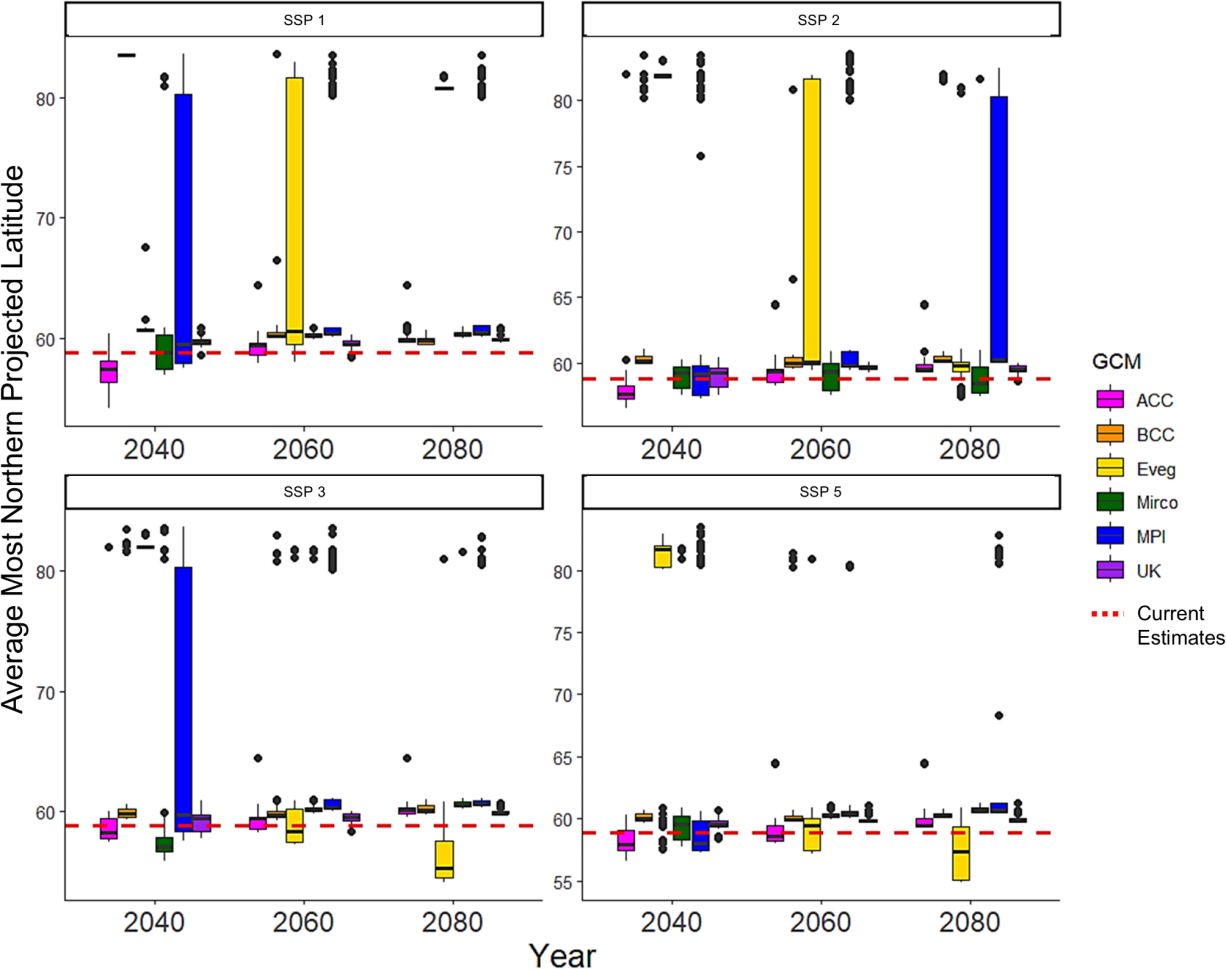
Global circulation model variability. Variation in most northern (i.e., maximum) projected suitable latitude for *D. rotundus* for each GCM across all four SSPs is shown across all three time periods. Current estimates of the most northern suitable latitude for *D. rotundus* are shown by a dashed red line. GCMS included: Australian Community Climate and Earth System Simulator (ACC, pink); the Beijing Climate Center Climate System Model (BCC, orange); the Europe wide consortium climate model (Eveg, yellow), the National Institute for Environmental Studies, University of Tokyo model (Mirco, green); the Max Planck Institute for Meteorology, Germany model (MPI, blue); and the U.K. Earth System Model (UK, purple). While almost all future projections are above current estimates, some GCMs had higher rates or variation in their estimation than others. The Europe wide consortium climate model (Eveg, yellow) and the Max Planck Institute for Meteorology, Germany model (MPI, blue), which both have low sensitivity to increased CO_2_ concentrations, had the highest rates of variation in their projections. GCMs are calibrated with differing metrics and effects of Earth system functions such as aerosol forcing^75,78,100^. For example, the MPI and EVeg models have relatively low values of aerosol forcing^75^, which could contribute to the uncertainty we identified.

Our analyses were conducted across an ensemble of six GCMs to capture a summary of current climate change projections. We found variation in the magnitude of climate suitability change for *D. rotundus* among the GCMs utilized (Fig. 6). For instance, latitudinal variation in *D. rotundus* ranges was influenced by the GCM used. While almost all models predicted northern expansion, some GCMs suggested higher expansion. For example, Eveg and MPI are GCMs with low sensitivity to increased CO_2_ concentrations and highest rates of variation in *D. rotundus* predicted ranges (Fig. 6). One possible explanation for this variance is that GCMs are calibrated with differing metrics and effects of Earth system functions such as aerosol forcing^75,78,100^. For example, the MPI and EVeg models have relatively low values of aerosol forcing^75^, which could contribute to the uncertainty we identified. Furthermore, ECS values for each GCM can be impacted by factors such as cloud feedback and cloud-aerosol interactions, which was identified in many GCMs between CMIP5 and CMIP6^75^. These factors can increase the range and value of ECS, which could have impacted the projection of our model across the GCMs^75,78^. Ultimately, our results indicate that the variation between GCM sensitivity and associated variance in projections should be taken into account when conducting ecological niche modeling under future climate change scenarios. Models predicting futures species distributions using a single GCM, could generate incomplete or misleading estimates of likely species ranges.

We chose to use non-extrapolated projections of our model to future scenarios to generate strict model transferences without prediction in novel climates^65,80,101^. Model transference without extrapolation is a conservative approach that does not assume fast evolutionary adaptation of species to climate change^81,102^. Furthermore, the use of only abiotic climatic variables in our model innately limits the local-level forecasting ability of our future projections of *D. rotundus* distribution at the population level. Nevertheless, the uncertainty present in future land-use projections can heavily impact future climate-change projections of temperature and precipitation, which are strongly influenced by urban and agricultural land-use changes^59^. We considered that inclusion of future landscape or land-use projections may have added more error than was useful for a continent-wide assessment. Biotic or movement based factors, such as prey density or shelter availability may drive *D. rotundus* presence and absence in areas predicted to be climatically suitable, making our climate-only models an overestimation of actual distributions, which has been termed ‘Eltonian Noise’^103–105^. Our results should therefore be viewed as an assessment of *D. rotundus*’ fundamental niche through which general VB-RABV mitigation strategies and more targeted studies can be planned in areas predicted to be suitable in the future for *D. rotundus*. Future research could also explore the possibility of *D. rotundus* adaptation to novel climates, which could refine distributional estimations once the landscape-level data become available.

## Conclusions

We found that the rabies reservoir *D. rotundus* could extend its range in the next 20–80 years into novel areas due to changes in climate. Areas suseptable to a future range expansion for *D. rotundus* include the southern US and south-central portions of Argentina and Chile. While certain areas may gain climate suitability in the higher latitudes of *D. rotundus* range, we found areas in the Amazon Rainforest may become unsuitable for this species in the future. Nevertheless, any loss of suitable area for *D. rotundus* may be offset by gains of suitability in areas that are currently acting as climatological barriers to dispersal for *D. rotundus* in the US and Argentina. Successful dispersal into new areas, however, would be limited by availability of resources, including prey density^86,96^. We recommend preventive and educational programming to be designed for areas predicted to be vulnerable to a *D. rotundus* range expansion. A likely range expansion for *D. rotundus* could result in increased human-wildlife conflict due to novel human and livestock populations exposed to *D. rotundus* invasion and possible exposure to VB-RABV.

## Electronic supplementary material

Below is the link to the electronic supplementary material.

Supplementary Material 1

## Data Availability

The datasets used and/or analyzed for this study are available from open access links at 10.6084/m9.figshare.15025296 and https://www.worldclim.org/data/cmip6/. Code for the analyses is available upon request from the corresponding author.
