# Supplementary material for: Future climate change and the distributional shift of the common vampire bat, Desmodus rotundus
Source: Sci Rep. 2025 Feb 18;15:5989. doi: 10.1038/s41598-025-87977-7 (PMC11836402; doi:10.1038/s41598-025-87977-7)
Supplement: Supplementary file 1 — Supplementary Material 1 [file 41598_2025_87977_MOESM1_ESM.docx]

**Supplementary Information: Future climate change and the distributional shift of the common vampire bat, *Desmodus rotundus***


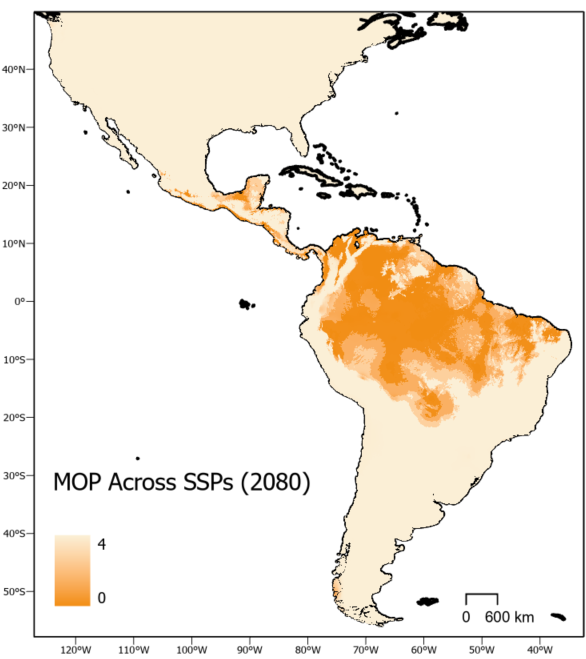


**Figure S1: Geographic patterns of strict extrapolation risk.** Post projection analysis of Mobility-Oriented Parity (MOP) for all four Shared Socioeconomic Pathways (SSPs one, two, three, and five) for the 2080 time period. An MOP analysis identifies areas with the most dissimilar climate conditions from the background environmental variables in the calibration data (i.e., where one or more covariate variables in the climates for projection have values outside ranges present in climates used for model calibration). Areas with similar climates are represented by zero values. Values above zero represent levels of climatic difference between the calibration area and the projected study areas across all SSPs (i.e., future scenarios). Areas with strict extrapolation risk, where novel climates are expected, are mainly located in the Amazon Rainforest and in some areas of Central America. Figure created using ArcGIS Pro version 2.5 software^66^.
